# Answer to Mr. Griffin's Queries on Vaccination, from Physicians and Surgeons of Edinburgh, Perth, and Dundee

**Published:** 1804-07-01

**Authors:** Wm. Farquharson, James Bryce, A. Gillespie

**Affiliations:** Surgeon to the Vaccine Institution at the Public Dispensary of Edinburgh. Edinburgh; Surgeon to the Vaccine Institution at the Public Dispensary of Edinburgh. Edinburgh; Surgeon to the Vaccine Institution at the Public Dispensary of Edinburgh. Edinburgh


### Answer to Mr. Griffin's Queries on Vaccination, from Physicians and Surgeons of Edinburgh, Perth, and Dundee.

*June* 15, 1804.

*We have received the following Communications from Dr. Walker, who has just obtained them from Mr. Griffin, passing through London last night on his way home from Scotland. The Rev. Gentleman, however, must pardon our bringing forward, in italics, what he, minister of peace, wished to be kept back for fear of any interruption of quiet and good neighbourhood. The Medical Council of the Royal Jennerian Society have voted him thanks for his pamphlet, and for his exertions to promote the diffusion of Vaccine Inoculation; and he must not conceal or curtail the valuable testimony of our northern neighbours. We understand, that if his stay had been longer in Edinburgh, he would have been favoured with the whole weight of that great school of physic in favour of the Jennerian discovery.*

To the Rev. Mr. GRIFFIN.

Sir,

OUR friend, Dr. Charles Stuart, has transmitted to us a letter which he received from you a few days ago; and has requested us to answer the following queries which were contained in it, viz.

Query 1. How long has vaccine inoculation been practised in this city or neighbourhood?

Query 2. Whether any facts have occurred tending to render it doubtful, whether the vaccine inoculation be a permanent preventive of variolous infection?

Query 3. Whether any have been evidently exposed to the infection of small-pox who have been vaccinated more than three years?

Query 4. Whether it be not the opinion of the medical gentlemen of Edinburgh, that if vaccination be a preventive for one year, it will be a preventive for life?

Answer

Answer to Query 1. Vaccination was begun here in the year 1799, and became general the following year.

On the 18th of February, 1801, we began to inoculate at the public dispensary, and since that time have vaccinated above three thousand, besides many hundred children privately. There must also have been a very great number vaccinated by the other medical practitioners, as they have all heartily concurred in their endeavours to convince the lower ranks of the propriety of having their children vaccinated; and they have succeeded much beyond their expectations.

Answer to Query 2. We have met with no facts to render it doubtful whether vaccination be a permanent preventive of variolous infection. Nor have we ever heard from our brethren, or any others, of such facts occurring in this city or neighbourhood.

Answer to Query 3. We know of many children, vaccinated more than three years ago, who have of late been repeatedly exposed to the infection of small pox; among others, some of our own children, who were vaccinated five years ago, without having suffered from the variolous infection. Indeed, we hear daily of such instances from the mothers of the children who were vaccinated at the dispensary early in the year 1801. Besides, the small-pox have been so frequent here for many months past that innumerable instances of the kind must have occurred, had the preventive not been permanent.

Answer to Query 4. We think that it must not only be the opinion of the medical gentlemen of Edinburgh, but of every thinking man, that if vaccination be a preventive of variolous infection for a year, it must be a preventive for life. The contrary opinion appears to us to be very unphilosophical, and contrary to our ideas of physiology and pathology. *Therefore we humbly think that Mr. Goldson's pamphlet is founded on such false principles, that no part of it can do any harm, except the* TITLE, *and that only to weak or unthinking people.*

We have thus answered your queries as fully as our time will admit of; and, although we have done it in our own names, we believe that we have given you the sentiments of all the medical practitioners of Edinburgh and the neighbourhood.

Sincerely wishing you success in your labours in the vineyard of humanity; and entreating that you may freely apply to us if you think that we can, in any way, assist you you in furthering the great work in which you are engaged, We are, &c.

WM. FARQUHARSON, M. D.
JAMES BRYCE,
A. GILLESPIE.
Surgeons to the Vaccine Institution at the Public Dispensary of Edinburgh.

*Edinburgh, June* 9, 1804.

*** Mr. Alexander Wood, Surgeon, Dr. Gregory, Dr. Charles Stroud, and, through him, Dr. Monro, have expressed their concurrence in the above to Mr. Griffin; also Drs. Stuart, Kelly, and Wood, of Perth, with Dr. Willis of Dundee, who have inoculated thousands, beginning more than four years ago, and from the first to the last exposing those whom they vaccinated to variolous contagion.

